# The Relationship between Body Image, Disability and Mental Health in Patients with Multiple Sclerosis

**DOI:** 10.3390/jcm12103606

**Published:** 2023-05-22

**Authors:** Viviana Lo Buono, Lilla Bonanno, Francesco Corallo, Davide Cardile, Giangaetano D’Aleo, Carmela Rifici, Edoardo Sessa, Angelo Quartarone, Maria Cristina De Cola

**Affiliations:** IRCCS Centro Neurolesi “Bonino-Pulejo”, S.S. 113 Via Palermo, C/da Casazza, 98124 Messina, Italy; viviana.lobuono@irccsme.it (V.L.B.); lilla.bonanno@irccsme.it (L.B.); davide.cardile@irccsme.it (D.C.); giangaetano.daleo@irccsme.it (G.D.); carmela.rifici@irccsme.it (C.R.); edoardo.sessa@irccsme.it (E.S.); angelo.quartarone@irccs.it (A.Q.); mariacristina.decola@irccsme.it (M.C.D.C.)

**Keywords:** multiple sclerosis, body image, disability, neuropsychiatric symptoms, self-esteem

## Abstract

Background: Multiple sclerosis is a progressive degenerative disorder that frequently involves the development of physical and emotional changes, including loss of limb function or sensitivity, sexual dysfunction, and cognitive and mood alterations. It is likely that these alterations lead to changes in body aspects. However, knowledge about body image perception in multiple sclerosis is lacking. Purpose: The present study investigated the relationship between body image perception and its correlation with a disability, neuropsychiatric symptoms, and self-esteem. Methods: A total of 100 outpatients with relapsing-remitting multiple sclerosis underwent neurological assessment using the Expanded Disability Status Scale. Participants also completed the Body Image Scale (BIS), Rosenberg Self-Esteem Scale (RSES), and Symptom Checklist-90-Revised (SCL-90-R). Results: We found a significant positive correlation between body image and disability (r = 0.21; *p* = 0.03), body image and self-esteem (r = −0.52; *p* < 0.001), body image and somatization (r = 0.44; *p* < 0.001), body image and depression (r = 0.57; *p* < 0.001), and body image and anxiety (r = 0.5; *p* < 0.001). Conclusions: The body is considered one of the main parts of a person’s identity. Dissatisfaction with one’s own body changes the general evaluation of the “self”. The body image construct has important health outcomes and should be studied more in patients with multiple sclerosis.

## 1. Introduction

Multiple Sclerosis (MS) is a chronic inflammatory demyelinating disease of the central nervous system [1]. The incidence and the prevalence of this non-traumatic disabling disease is increasing both in developing and developed countries [2]. Although the actual causes are still unknown, it has been seen that lifestyle (obesity, smoking) coupled with environmental factors such as exposure to ultraviolet B light or Epstein–Barr virus infection exposes individuals who have a genetic predisposition to be susceptible to the disease particularly at risk [3,4,5].

There are different forms of multiple sclerosis; these forms differ in the course of the disease and the way they affect the neurological level.

According to the course of disease, patients may be subdivided in four categories: primarily progressive, secondarily progressive, progressive-relapsing, and relapsing-remitting MS. Primary progressive multiple sclerosis (PPMS) is characterized by a slow deterioration of neurological functions from the onset of the first symptoms and the absence of major ‘attacks’ and substantial moments of remission. The secondary progressive form (SMPS) consists in a constant worsening of neurological functions and a progressive accumulation of disability in the absence of notable moments of remission [6]. Progressive-relapsing form (PRMS) involves a constant deterioration of neurological functions from the very beginning and is characterized by major attacks and the absence of moments of remission. Finally, the relapsing-remitting multiple sclerosis is the most frequent form, occurring in about 85% of cases (at the onset), and is characterized by acute episodes of neurological deficits (relapses) followed by partial or total regression of symptoms [1]. However, a residual deficit often persists after a relapse, leading to a gradual increasing of disability during the disease.

Multiple sclerosis has historically been classified as an organ-specific T-cell mediated autoimmune disease. However, the success of B-cell targeted therapies challenges the standard T-cell autoimmune dogma [7]. It is traditionally viewed as a two-stage disease, with early inflammation responsible for relapsing-remitting disease and delayed neurodegeneration causing non-relapsing progression, i.e., secondary and primary progressive MS [8,9].

The emergence of increasingly effective biological therapies and an active approach to treating MS, in particular treating to a target of no evident disease activity (NEDA), are changing the long-term outcome for people with MS (pwMS). More aggressive immune reconstitution therapies that result in a proportion of pwMS entering long-term remission offer a small number of pwMS a potential cure [10]. Recent positive trials of disease-modifying therapies in progressive MS offer those with more advanced MS the hope of slowing their disease progression, with preservation of residual function [11]. The fact that treatments appear to work at multiple stages in the disease course significantly challenges the traditional two-stage view of the natural history of MS [12].

Body image refers to the emotional attitudes, beliefs, and perceptions people have about their own bodies. It is the mental representation an individual creates of himself/herself based on experiences, concepts, and behaviors and includes an emotional view of his/her body [1]. We are conscious of our own body through the experience of its physicality but also of the different neural representations that are related to the subjective experience [13]. The real or distorted body image representation can be influenced by emotional experience [14], and it seems to be related to self-esteem [15].

Chronic diseases associated with brain alterations, physical pain, and psychological distress can cause disturbances in various components of self-concept and loss of self and identity [8]. Each time modifications occur in the body, the psychological perception of the body has to be modified as well and adapted in the context of changing physical conditions [9].

There is an increasing amount of research on body image in chronic diseases including cancer and rheumatic diseases. Body dissatisfaction refers to the negative evaluation of one’s body and indicates a discrepancy between a person’s ideal body and perceived body [10]. This distortion of body perception is a risk factor for the development of mental disorders, including disordered eating behaviors, depression, and low self-esteem [11]. Moreover, poor body image can influence competence in social and occupational functioning [14]. The experience of one’s own body is mediated by perceptual information, and it requires the processing and integration of several body signals in the premotor, temporoparietal, posterior parietal, and extrastriate cortex [15].

Regarding multiple sclerosis (MS), there have been few reports on the experience of body image changes, and most of them concentrate only on sexuality [10]. Some authors have reported altered multisensory integration of body signals that seem to interfere with mental body representation in SM patients [16]. Samonds and Cammermeyer [17] suggested a relationship between body image and the severity of physical disability. Indeed, physical, emotional, and cognitive changes might occur in MS patients that affect body image—that is, the way an individual perceives thinks or feels about his or her body [18]. In particular, the impact of the disease on the physical dimension (disability, pain, difficulty with balance) could lead to the formation of a new body image that, when associated with low self-perception, would reduce functioning in activities of daily living, worsen the quality of life [19], and increase psychological distress [20].

Other literature data have described the influence of mood alterations, particularly depressive symptoms, in the perception of altered body image [16]. Similar data were found by Sengul et al. [21] who added among the causes of body dissatisfaction the following: older age, being single/divorced or widowed, early onset of illness, and relapses frequency. Ageing and the physiological transformation of one’s body during the disease phase generates great stress in patients, especially in female patients [22]. However, the incidence of altered body image reported by MS patients is still unclear [23,24], and it is not yet known whether this pathological process is associated with the disease.

Given the importance of body image in many pathologies and given the lack of evidence in literature, in this retrospective study, we analyzed the perception of body image in MS patients and correlated it with the severity of a physical disability, neuropsychiatric symptoms, and self-esteem.

## 2. Materials and Methods

### 2.1. Study Design

This is a retrospective cross-sectional study including a sample of patients referred to the multiple sclerosis outpatient clinic of IRCCS Centro Neurolesi “Bonino-Pulejo” in Messina, Italy, over the course of two years. This retrospective cohort study did not require the approval of the Ethics Committee, in accordance with the current rules of our hospital. However, all participants were contacted and provided written informed consent to enter the study.

### 2.2. Study Population

One hundred patients with relapsing-remitting multiple sclerosis were enrolled (26 men, 74 women) aged from 18 to 67 years. The only inclusion criterion for selection was having a diagnosis of MS according to the 2017 revisions to the McDonald Criteria, whereas exclusion criteria were (i) history of major psychiatric disorders and (ii) other neurological disorders.

### 2.3. Assessment

During the examination, an experienced neurologist made the diagnosis of MS according to the McDonald criteria revised in 2017 and evaluated the disease’s disability by means of the Expanded Disability Status Scale (EDSS) [25]. Moreover, participants underwent the Body Image Scale (BIS) [26], the Rosenberg Self-Esteem Scale (RSES) [27], and the Symptom Checklist-90-Revised (SCL-90-R) [28]. The BIS is a 10-item questionnaire used to assess various dimensions of body image. Each question is rated on 3 points Likert scale (0 = not at all; 3 = very much). The final score is the sum of the 10 items, ranging from 0 to 30, with a zero score representing the absence of symptoms or distress and highest scores representing increasing symptoms and distress or more body image concerns.

The RSES is a Likert-type scale that measures global self-worth by measuring both positive and negative feelings about the self. The SCL-90-R is a multi-dimensional self-report inventory, consisting of 90 items covering nine main psychological dimensions: somatization (SOM), obsessive-compulsive (OC), interpersonal sensitivity (IS), depression (DEP), anxiety (ANX), hostility (HOS), phobic anxiety (PHOB), paranoid ideation (PAR), psychoticism (PSY); and three global indices of distress: global severity index (GSI), positive symptoms total (PST), and positive symptom distress index (PSDI). In addition, from the hospital medical records, the type of disease-modifying therapies (i.e., IFNβ-1a, glatiramer acetate, fingolimod, ocrelizumab, dimethyl fumarate, natalizumab, teriflunomide) was extracted.

### 2.4. Statistical Analysis

Since both the Shapiro–Wilk test and the graphical inspection showed a not-normal distribution for most of the target variables, a nonparametric analysis was performed. Therefore, continuous variables were expressed as median and first-third quartile, whereas categorical variables were expressed as frequencies and percentages. The χ2 test with continuity correction or the Fisher’s exact test were used to assess for statistical differences in proportions, when appropriate, whereas the Mann–Whitney U was used to compare continuous variables. Correlations were computed by Spearman’s coefficient. Finally, nonparametric Poisson regression was performed (by using the MASS package of R) to estimate the effects of patients’ psychiatric symptoms (Independent variables) on body image/self-esteem (dependent variable), after adjustment for age, EDSS, and disease duration. The backward elimination stepwise procedure for the choice of the best predictive variables according to the Akaike information criterion (AIC) was applied. As a measure of Goodness-of-Fit, we computed a Chi-squared test with degree of freedom equal to the number of predictors in the model. Analyses were performed using an open-source R4.0.5 software package, considering a *p <* 0.05 as statistically significant.

## 3. Results

The study included patients aged from 18 to 67 years, and about 74% were females. No significant gender differences were found, except for the type of medication taken for MS treatment (χ^2^(7) = 24.99; *p* < 0.001). A detailed description of the sample is reported in Table 1.

**Figure 1:**
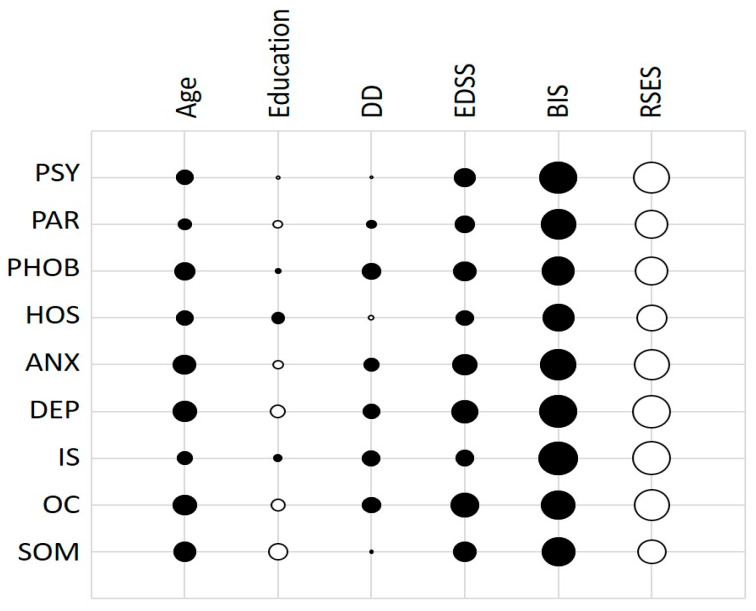
Correlations between the psychological dimension of SCL-90 and clinical and demographical data.

We found several strong correlations between both body image and patients’ self-esteem and psychopathological symptoms, as shown in Figure 1. Notably, the strongest correlations were between BIS and IS (r = 0.60), BIS and DEP (r = 0.57), BIS and PSY (r = 0.55), and between RSES and IS (r = −0.60), RSES and DEP (r = −0.59), RSES and PSY (r = −0.55). When we correlated BIS and RSES, we found that both strongly correlated with GSI (r = 0.58 and r = −0.56, respectively) and PST (r = 0.57 and r = −0.62, respectively) and moderately with PSDI (r = 0.34 and r = −0.20, respectively).

### Poisson Regression Analysis

As shown in Table 2, EDSS and age were significant predictors of body image (*p* < 0.0001 and *p* < 0.001, respectively), as well as all three SCL-90-R global distress indices. Notably, the sign of coefficients would seem to indicate that the functional disability and the severity of psychopathological symptoms might be risk factors for the development of distress concerning body image in SM patients. On the contrary, only PST was a significant predictor of self-esteem (*p* < 0.0001). In addition, we found that all dimensions of the SCL-90-R scale were significant predictors of both body image and self-esteem (Table 3), confirming the results of the previous correlation analysis.

## 4. Discussion

This study explored the body image perceptions in people with MS and its relationship with the severity of the physical disability, psychopathological symptoms, and self-esteem. In agreement with other authors [17], the degree of disability was an important risk factor for the development of greater body image-related distress. Physical disability has a negative influence on people’s psychological experience, attitudes, and feelings about their own bodies. In MS, the progression of the disease with the consequent loss or alteration of motor functions, sensory and cognitive, seems to cause an increase in psychopathological symptoms related to the possible loss of autonomy and fear of diminishing the reliability of one’s body. Results showed also that body image perception and self-esteem were linked with the presence of depressive symptoms, feelings of personal inadequacy and inferiority in comparison with others, manifestations of paranoid thinking, hostility, and suspiciousness. In addition, both body image perception and self-esteem were correlated with being affected by the intensity of the psychic discomfort as complained by the subjects. Negative perceptions about themselves usually are associated with negative emotion regulation strategies and dysfunctional coping [29]. Murray et al. [30] have investigated the association between body image and emotion regulation as one of the significant factors of mental health. Hughes and Gullone [31] have described a feeling of inferiority related to several emotions such as anger, depression, and stress, and it affects the quality of life in subjects with a negative self-image. Emotion regulation is a key factor in determining well-being and successful performance and plays an important role in coping with stressful events such as chronic disease [32]. As we have already mentioned, disease severity certainly plays a role in body perception, but that is not all. A study by Pfaffenberger et al. [8] reveals how the patient’s concerns also play a role in the body’s perception in the course of illness. RRMS patients even with mild disability reported high worries about physical deficits. Specifically, male patients were more concerned about problems related to the sphere of sexuality, whereas female patients were more worried about changes related to the physical sphere and the fear of gaining weight and becoming less attractive. These results would also seem to call into question the strong stigmatization of body weight in connection with disability and body image. If physiological ageing occurs during concomitant pathology, the relationship with one’s own disability becomes less comfortable [33], and the sense of discomfort with external prejudice increases.

Body image is a dynamic concept corresponding to a sort of internal and innate figure that the individual creates for him/herself in the mind. It is a mental representation that treats the bodily experience from a certain point of view, but also and above all, a cognitive, affective, and metacognitive point of view that can, to a great extent, determine the self-esteem of the individual [34,35]. MS is one of the most prevalent chronic diseases of the central nervous system, which has a disabling nature [36]. Body image perception has not given rise to much debate in SM although body representations are subject to complex distortions due to disease [16]. MS affects, indeed, many spheres of functioning associated with physiological and psychological change that reduces physical function and leads to disability with a substantial impact on the sense of identity and redefinition of self-image [37]. In addition, individuals with MS are often plagued by the unpredictability of their disease and have to contend with uncertainty in their life and significant life changes. This can lead to high levels of stress, perceived lack of control, helplessness, depression, and anxiety that can influence their own body image [38,39].

The experience of a healthy body fundamental to health is characterized by harmony, the possibility of control, and predictability. The body in the experience of illness is generally qualified as negative and characterized by objectification, alienation, and perturbation [40]. For people with MS, one’s own body may no longer be taken for granted but may become instead a disturbing presence [41]. 

Numerous factors interact in the complexity of the experience of illness and in the perception of the body image: aspects of premorbid personality, coping strategies, and affective-emotional characteristics. MS leads, therefore, to rethinking the meaning of life, and poor body image can affect physical and psychological health and can influence self-esteem, mood, competence, social functioning, and occupational functioning. Body image perception is difficult to measure. However, because of the strong links between body image alteration and poor health outcomes, it is important for clinicians to be able to determine risk factors in patients that may predispose them to this disorder. For this reason, it might be useful to evaluate body image perception in MS daily practice. 

Future studies should include larger sample sizes to reduce the possibility of lower power contributing to a lack of significance and to develop measuring instruments specifically for people with MS. Moreover, since body image changes with aging, it could be interesting to evaluate this change in clinical populations and to compare it with a healthy group. In addition, future research could also explore the effectiveness of psychotherapeutic or pharmacological interventions targeted at reducing depression and anxiety and at improving body image and, consequently, other health outcomes in MS patients.

## Figures and Tables

**Table 1:** Demographic and clinical description of the sample.

Characteristics	All (n = 100)	Males (n = 26)	Females (n = 74)	*p*-Value
Age (years)	40.3 ± 11.6	41.8 ± 11.0	39.8 ± 11.8	0.99
Education (years)	12.9 ± 3.2	13.0 ± 3.6	12.9 ± 3.1	0.99
Disease duration	10.0 ± 7.4	10.0 ± 7.8	10.0 ± 7.4	0.99
EDSS score	2.9 ± 1.6	3.4 ± 2.0	2.7 ± 1.4	0.99
Drug for MS				<0.001
None	6 (6.0)	4 (15.4)	2 (2.7)	
Natalizumab	49 (49.0)	10 (38.5)	39 (52.7)	
IFNβ-1°	15 (15.0)	2 (7.7)	13 (17.6)	
Dimethyl fumarate	7 (7.0)	1 (3.8)	6 (8.1)	
Teriflunomide	7 (7.0)	4 (15.4)	3 (4.1)	
Fingolimod	6 (6.0)	1 (3.8)	5 (6.7)	
Glatiramer acetate	6 (6.0)	0 (0.0)	6 (8.1)	
Ocrelizumab	4 (4.0)	4 (15.4)	0 (0.0)	

Continuous variables were expressed as mean ± standard deviation, whereas categorical variables as frequencies and percentages. Significant differences are in bold. EDSS = Expanded Disability Status Scale.

**Table 2:** Poisson regression results including SCL-90-R global distress indices were a predictor for body image and self-esteem after backward stepwise selection.

Dependent Variable	Coefficients					*p* (Model)
	Predictors	Estimate	Std. Error	z Value	*p* Value	
BIS	EDSS	0.116	0.023	4.968	<0.0001	<0.0001
	Age	−0.012	0.003	−3.860	<0.001	
	GSI	−0.023	0.010	−2.339	0.019	
	PSDI	0.023	0.007	2.999	0.003	
	PST	0.057	0.012	4.668	<0.0001	
RSES	PST	−0.012	0.002	−6.698	<0.0001	<0.0001

LEGEND: EDSS = Expanded Disability Status Scale; BIS = Body Image Scale; RSES = Rosenberg Self-Esteem Scale; GSI = Global Symptom Index; PSDI = Positive Symptom Distress Index; PST = Positive Symptom Total; *p* (Model) = the *p*-value is the statistical significance of general model.

**Table 3:** Poisson regression results: each model included one psychological dimension of the SCL-90-R as predictor for body image or self-esteem. Confounder estimates (age, EDSS, and disease duration) are not reported.

Dependent Variable	Coefficients					*p* (Model)
	Predictor	Estimate	Std. Error	z Value	*p* Value	
BIS	SOM	0.014	0.002	8.219	<0.0001	<0.0001
	OC	0.015	0.002	8.861	<0.0001	<0.0001
	IS	0.019	0.002	11.981	<0.0001	<0.0001
	DEP	0.021	0.002	11.740	<0.0001	<0.0001
	ANX	0.015	0.001	9.577	<0.0001	<0.0001
	HOS	0.012	0.002	7.359	<0.0001	<0.0001
	PHOB	0.013	0.001	9.811	<0.0001	<0.0001
	PAR	0.020	0.002	11.130	<0.0001	<0.0001
	PSY	0.015	0.001	11.402	<0.0001	<0.0001
RSES	SOM	−0.003	0.001	−2.743	0.006	<0.001
	OC	−0.006	0.001	−5.470	<0.0001	<0.0001
	IS	−0.010	0.001	−6.751	<0.0001	<0.0001
	DEP	−0.009	0.001	−6.435	<0.0001	<0.0001
	ANX	−0.006	0.001	−5.268	<0.0001	<0.0001
	HOS	−0.004	0.001	−3.510	<0.0001	<0.0001
	PHOB	−0.006	0.001	−5.276	<0.0001	<0.0001
	PAR	−0.005	0.001	−3.801	<0.0001	<0.0001
	PSY	−0.006	0.001	−5.875	<0.0001	<0.0001

LEGEND: somatization (SOM), obsessive-compulsive (OC), interpersonal sensitivity (IS), depression (DEP), anxiety (ANX), hostility (HOS), phobic anxiety (PHOB), paranoid ideation (PAR), psychoticism (PSY), Body Image Scale (BIS), Rosenberg Self-Esteem Scale (RSES). *p* (Model) is the *p*-value is the statistical significance of general model.

## Data Availability

The data that support the findings of this study are available on request from the corresponding author.
